# Beyond diagnosis in psychiatric practice

**DOI:** 10.1186/s12991-020-00279-2

**Published:** 2020-04-15

**Authors:** Mario Maj

**Affiliations:** Department of Psychiatry, University of Campania L. Vanvitelli, Largo Madonna delle Grazie, 80138 Naples, Italy

**Keywords:** Diagnosis, Clinical characterization, Precision psychiatry, Dimensions, Severity, Clinical staging, Antecedent variables, Concomitant variables

## Abstract

In psychiatry, the information conveyed by diagnosis (i.e., the “type” to which the individual patient is reconducted) is in itself insufficient for therapeutic and prognostic purposes. Hence the need for a more detailed characterization of the individual case, with a special focus on the assessment of low-order and high-order psychopathological dimensions, the evaluation of the severity of the clinical picture, the assessment of the stage of development of the disorder, and the exploration of a series of antecedent and concomitant variables. We should start to promote the construction and validation of tools guiding the clinician systematically in this characterization, trying to incorporate in this effort elements of the approaches that are currently presented as “alternative” to the ICD and DSM.

## Introduction

In psychiatry, more than in other medical specialties, diagnosis (i.e., the act of reconducting the individual case to a “type”, among those included in a classification system) is in itself insufficient to guide the formulation of a tailored management plan and a reliable prognosis.

While in other medical specialties diagnosis brings with itself a quantum of information which points also to a given etiology and/or pathogenesis, and therefore informs in a significant way the choice of the therapeutic intervention, the quantum of information that diagnosis brings with itself in psychiatry is somewhat lower [1].

Indeed, both research evidence and clinical experience tell us that patients sharing the same psychiatric diagnosis often respond differently to a given treatment, while patients with different psychiatric diagnoses may respond similarly to a given treatment (not to mention the wide variability of outcomes in people receiving the same diagnosis).

Several other medical specialties are currently pursuing “precision medicine” [2], i.e., a more precise characterization of the individual case beyond diagnosis, aiming to make the management plan more tailored. In this “precision medicine”, biological markers are intended to have an important role. In psychiatry, we do not have biological markers ready for use in ordinary clinical practice, but we need even more—for the reasons that we have just seen—a further characterization of the individual case beyond diagnosis. How can we deal with this situation?

The answer is that our “precision psychiatry” has today essentially a clinical foundation. It consists in the evaluation of the individual case to whom we have applied a given diagnosis with respect to a series of clinical variables.

In the past 50 years, diagnosis has attracted a lot of attention in psychiatry, with the development and continuing refinement of several diagnostic systems and diagnostic interviews, whereas the need for the further characterization of the individual case has been largely ignored. This has generated an inter-clinician variability in the implementation of this latter step which is not less significant and deleterious than that described for the step of diagnosis in the 1970s, which brought to the introduction of the DSM-III operational diagnostic approach.

The focus on diagnostic categories in most research and in virtually all treatment guidelines, as well as the priority given to pharmacological interventions, for which a simplistic and stereotyped relationship between “diagnosis” and “treatment” can be more easily proposed, has certainly contributed to this situation. The emphasis on the “equivalence” rather than on the differences between the various treatments in controlled trials has been a further element leading to the current oversimplification of clinical practice (i.e., virtually all medications and all psychotherapies for a given mental disorder are now often regarded as interchangeable, which obscures the need for a more detailed characterization of the individual case).

Several approaches that are currently presented as “alternative” to the ICD and the DSM—such as those focusing on psychopathological dimensions [3–5] or networks [6, 7], or those assuming that the neurobiological underpinnings of psychopathology should be the major drivers of psychiatric classifications [8, 9] —are actually unlikely to “replace” in the future our current diagnostic practices, but may be able to improve significantly the further characterization of the individual case, thus complementing current diagnoses.

What are, or may be, in fact, the main elements of this detailed characterization of the individual case? Let us summarize them in the following sections.

### Dimensional characterization

A first element in the characterization of the individual case is its evaluation with respect to the psychopathological dimensions relevant to the diagnosis that has been made. This dimensional assessment can actually be implemented at two levels.

The first level is already very clear to the average clinician. It consists in the identification, within the syndrome that has been diagnosed, of a series of components (e.g., in the case of psychoses, the positive, negative, disorganization, depression, manic and cognitive dimensions).

In ordinary clinical practice, the importance to assess the individual case who has received a diagnosis of psychosis with respect to each of the above dimensions is increasingly acknowledged. There is, however, an unmet need for an instrument which can guide the clinician systematically in this dimensional evaluation. The DSM-5 provides some indications in this respect in one of its appendices, but they appear somewhat simplistic and of scarce clinical utility.

On the other hand, the rating scales commonly used in research on the dimensions of psychoses do not seem to be suitable for adoption in ordinary clinical practice, because of the time they take for administration, the training they require for appropriate use, and the lack of evidence of interrater reliability in routine settings. The recent attempt to develop and validate assessment tools for the negative dimension of schizophrenia that are simpler to use in clinical contexts is notable in this respect [10].

The second level of the dimensional characterization, much less clear at the moment to the average clinician, manifests itself in an exemplary way in the case of depression. We have been aware for decades that, especially in primary care settings, depression is often accompanied by an anxiety and a somatic component. A possible conceptualization of this reality is that in these instances the depressive syndrome is part of a high-order dimension, called *distress* or *internalizing* [11, 12].

The characterization of the individual case of depression with respect to this high-order dimension is important, because it can lead to the choice of a transdiagnostic psychotherapeutic intervention, having as a target not only depression, but also the other two components. This kind of interventions is today available, especially on the cognitive–behavioural side [13].

However, in other cases, more frequently in men than in women and in adolescents than in adults, the depressive syndrome may instead be accompanied by anger, aggression, substance abuse, and risk-taking behaviours [14]. In other terms, it may be part of a different high-order dimension, that can be defined as *externalizing* [15]. The implications of this other dimension for the formulation of the management plan are obviously very different from those of the *distress* dimension.

Thus, characterizing the individual patient with a diagnosis of depression with respect to these high-order dimensions is important for the formulation of the therapeutic plan. We lack at the moment an awareness of the importance of these dimensions in the psychiatric community, and we do not have validated instruments for their assessment in ordinary clinical practice. We expect the ongoing project named Hierarchical Taxonomy of Psychopathology (HiTOP) [4, 16], usually presented as a possible alternative to our current diagnostic systems, to help us define more clearly these high-order dimensions and to develop instruments allowing us to assess them reliably in ordinary clinical conditions.

### Severity

The severity of the clinical picture is a further important element for the formulation of the management plan [17]. This is particularly acknowledged today in the case of depression, since treatment guidelines propose different therapeutic approaches depending upon the severity of the current depressive episode [18, 19].

It is a fact, however, that both the ICD and the DSM provide definitions of mild, moderate and severe depression that are too generic, not evidence-based, and indeed very rarely used in ordinary clinical practice. These definitions are also ignored in trials of medications and psychotherapies, in which the global score on a rating scale for depression is commonly used to assess severity [20]. However, recent studies based on the “network approach” have documented that the practice of adding up the scores for individual depressive symptoms is most probably inappropriate, because the various symptoms have a different weight in determining the severity of a depressive episode [21].

There is, therefore, an unmet need for a tool assessing the severity of depression in ordinary practice. It is our auspice that the network approach, rather than being proposed as “alternative” to current diagnostic systems, will help us evaluate the severity of depression and other psychiatric syndromes in a way which is more reliable and useful for the formulation of the management plan.

### Clinical staging

A third element to be considered in the clinical characterization of the individual case with a given diagnosis is the stage of the disorder, i.e., the phase in the evolution of the pathology in which the patient currently is. This is obviously important for the formulation of the management plan.

We have now staging systems for psychoses [22], bipolar disorder [23] and depression [24], some of which have been validated for use in clinical practice. In these systems, the therapeutic implications of each stage are usually made explicit. It is acknowledged that the progression from a stage to the following one is not irreversible, especially if the patient is properly treated. In the case of depression, systems are available also for the staging of “treatment-resistant” depression [25].

An aspect which is common to these staging systems is the identification of a first stage, sometimes named *stage* 1*a*, marked by a symptomatology that is described as fluid, aspecific, undifferentiated, to which only in a later stage (*stage* 1*b*) a specific, although subthreshold, symptomatology is added (*ultra*-*high risk stage* or *attenuated psychosis syndrome* in the case of psychoses) [26].

The identification of this first stage is important from the viewpoint of public health care, because it has led in some countries to the development of *soft entry points*, i.e., places well integrated in the community and separated from mental health departments, with a strong involvement of young volunteers, in which the declared objectives are socialization, educational and vocational support, and promotion of physical and mental health. The idea is to reach, through these soft entry points, the highest possible number of young people who are currently in the *stage* 1*a*, in order to refer timely those who start to manifest a more specific subthreshold symptomatology to the appropriate clinical services (e.g., centers for first psychotic episodes) [27].

### Antecedent variables

One more group of elements to be considered in the characterization of the individual case which has received a given diagnosis is represented by several antecedent variables.

A first antecedent variable to be explored is family history. For instance, a family history of bipolar disorder in a patient with depression is an information with important clinical implications. In a psychotic patient, knowing that there is another relative who is also psychotic, and collecting information about the treatment he/she has received and his/her response to that treatment, may be important. This assessment of family history is currently not implemented routinely in clinical practice, also because of the lack of an appropriate assessment tool validated for use in the various settings.

Further antecedent variables to be explored, especially in patients with diagnosis of psychosis, are perinatal history (e.g., a history of obstetric complications) and the history of psychomotor development and premorbid social adjustment in that person (which may be useful for the formulation of the rehabilitative intervention, especially on the side of cognitive remediation). Once again, these are neglected variables whose exploration is not currently guided by validated assessment instruments.

Also important is the history of early environmental exposures. For instance, a history of childhood physical or sexual abuse is a predictor of a poor response to currently available treatments in persons with depression [28]. This aspect is rarely explored in ordinary clinical conditions, also because we currently miss an assessment tool helping us to assess it in a systematic way, identifying false positives (not rare in the case of depression, because depressed people tend to reconstruct their past in a negative light).

Finally, we should consider psychopathological antecedents. For instance, in a patient with a current diagnosis of schizophrenia, a history of attention-deficit/hyperactivity disorder or conduct disorder during childhood or early adolescence is a predictor of a poor response to current antipsychotic medications [29]. This is a notion shared by very few clinicians.

### Concomitant variables

In a patient in whom we have made a given diagnosis, we should also consider a series of concomitant variables.

Cognitive functioning in the domains relevant to that particular syndrome is an important element for the formulation of the psychosocial intervention and increasingly also of the pharmacological one (not only in psychoses, but also in depression). Especially for patients with psychoses, we lack at the moment a well-validated battery of cognitive tests suitable for use in ordinary practice [30].

Very important is also the characterization of the social functioning in its various domains, not only on the side of weaknesses and disabilities, but also on that of strengths and resources, because both of them are relevant for the formulation of the psychosocial intervention. Some validated instruments for the assessment of social functioning are now available, but they are not well known and rarely used in ordinary practice [31].

An area neglected up to now in psychiatry, but which is emerging as significant, is that of positive emotions and well-being. In a depressed patient, the attenuation or suppression of negative emotions as an outcome of treatment does not necessarily imply the emergence of those positive emotions that the person is looking for. Up to some years ago, a common tenet was that the most important outcome of depression treatment is the reduction of symptoms from the viewpoint of clinicians, and the improvement of functioning at work, within the family and in interpersonal relationships from the viewpoint of the patient.

Today we know that this may not be exactly the case. The real priority from the viewpoint of many patients seems to be the recovery of positive emotions (well-being, the sense of meaning of one’s existence, the ability to enjoy the little pleasures of ordinary life). We have now assessment instruments which allow us to evaluate these aspects, and also some psychotherapeutic interventions helping us to promote them [32, 33]. Of note, some pharmacological treatments that are effective on depressive symptoms seem to produce at the same time an “emotional blunting” [34].

Essential is also the characterization of the patient with respect to substance abuse, which represents in itself a target for intervention, and affects the adherence and response to the therapies for the syndrome we have diagnosed. Moreover, we cannot ignore anymore today that physical comorbidities must represent a target for specific interventions, especially in psychotic patients, and have a significant impact on the choice of medication [35, 36].

## Discussion

It is with respect to the assessment of the above variables that clinicians need today a systematic guidance, which current diagnostic systems and related tools do not provide, or do not provide satisfactorily, thus contributing to a therapeutic practice which, being guided just by diagnostic labels, is often oversimplified and stereotyped.

The approaches that are currently regarded as “alternative” to the ICD and DSM may not turn out to be, in the future, a basis for a clinically useful reclassification of psychopathology. However, elements of those approaches may be increasingly incorporated in the further characterization of the individual case, which is at least as important as the application of a diagnostic label for the management choices and the formulation of prognosis.

We still need current diagnostic categories, which can certainly be much improved, but without which we would either be lost in a *mare magnum* of variables, or presented with synthetic formulations which are less efficient, in addition to being potentially controversial and not rooted in clinical tradition [1]. However, we should be aware that those categories are intrinsically insufficient to pursue the “clinical utility” objective of our current classification systems [37–39], because the act of diagnosis is only one step in the process leading to the optimal formulation of the management plan (especially if this does not include only the selection of a medication) and the prediction of outcomes (especially if this is meant to cover not only clinical variables, but also elements concerning social functioning and personal recovery).

We should start to promote the construction and validation of tools guiding the clinician systematically in the characterization of the individual case who has received a given diagnosis, with a special focus on the assessment of low-order and high-order psychopathological dimensions, the reliable evaluation of the severity of the clinical picture, the assessment of the stage of development of the disorder, and the exploration of a series of antecedent and concomitant variables.

We should try to incorporate in this effort elements of the approaches that are currently presented as “alternative” to the ICD and DSM, that is: (a) those focusing on psychopathological dimensions, either at the level of the entire domain of psychopathology (e.g., the HiTOP) [4, 5] or at the level of specific areas of psychopathology (e.g., the “transdiagnostic psychosis spectrum model”) [3]; (b) those presenting the entire domain of psychopathology as consisting of networks of symptoms and signs [6, 7]; and (c) those looking for the neurobiological underpinnings of mental disorder, once again either at the level of the entire domain of psychopathology (e.g., the Research Domain Criteria, RDoC) [8, 9] or at the level of specific areas of psychopathology (e.g., the neurodevelopmental gradient model) [40].

Since a detailed clinical characterization of the individual case with a given diagnosis may not be feasible in some contexts, because of time and resource constraints, a hierarchy of variables to be considered in the different clinical settings may need to be proposed. Furthermore, the fact that current research on psychiatric treatments does not seem to indicate a different efficacy of the various interventions on the different varieties or subtypes of currently identified syndromes should not be a deterrent to the above-mentioned developments. Indeed, what we are facing today is to a large extent a vicious circle (i.e., regulatory agencies require the documentation of the “equivalence” of any new treatment with a consolidated one; drug companies are keen to document the “equivalence” of their new products with the best selling ones; proponents of the various psychotherapies are keen to demonstrate that their interventions are “equivalent” to medications; and the proponents of each individual psychotherapy are keen to demonstrate that the intervention they put forward is active in all cases of a given syndrome, independently from varieties or subtypes).

Furthermore, the clinical characterization of patients recruited for clinical trials is usually very limited, and several varieties of the syndrome being considered are eliminated when fixing exclusion criteria. In this scenario, the “equivalence” between the various interventions is likely to emerge, whereas the differences are likely to be obscured. In spite of this, when secondary analyses of large databases—through individual patient data meta-analyses or machine learning—are performed, differences in the profiles of patients responding to different interventions (e.g., antidepressants vs. cognitive behavioral psychotherapy, or different classes of antidepressants in depression) are already emerging [41, 42].

## Conclusion

The spreading of a new culture focusing on the detailed characterization of patients sharing a given diagnosis is likely to break the above vicious circle, making our treatments less stereotyped. Hopefully, it will also reduce the proportion of patients with a given syndrome that are currently classified as “treatment-resistant” [43] simply because they have received interventions that, although validated for that syndrome, are suboptimal for that individual case.

## Data Availability

Not applicable.
